# Will Gene Editing Orphanize Rare Diseases?

**DOI:** 10.1016/j.jacbts.2022.12.002

**Published:** 2023-01-23

**Authors:** Douglas L. Mann

The presentation by Julian Gillmore and colleagues at the 2022 American Heart Association meeting, which showed that a single intravenous infusion of NTLA-200, a novel CRISPR/Cas9 gene-editing technology, significantly reduced abnormal levels of transthyretin protein in patients with ATTR amyloid cardiomyopathy, ushered in a new era of cardiovascular therapeutics.^1^ Although the concept of using gene editing to treat cardiovascular disease seemed like a remote possibility just a few years ago, the progress in the field of gene editing has been striking. Two biotechnology companies, CRISPR Therapeutics and Vertex, have used gene editing to prevent recurrent vaso-occlusive crisis in patients with sickle cell disease. Verve Therapeutics is developing CRISPR therapeutics directed against proprotein convertase subtilisin/kexin type 9 and angiopoietin-like 3 protein, which are 2 proteins that govern lipid metabolism. Although these CRISPR-based therapies are potentially disruptive, insofar as they may replace the chronic care model for atherosclerotic cardiovascular diseases with a single infusion of a gene-editing therapeutic, they are also poised to disrupt the health care budget, and possibly create inequities in health care for countries that cannot afford these new therapies.

Although pricing for gene editing strategies has not been established. based on the pricing for current gene therapies, which range from $435,000 for a single intraocular injection of voretigene neparvovec to treat vision loss secondary to inherited retinal dystrophy to $3.0 million dollars/dose to treat the rare neurological disorder cerebral adrenoleukodystrophy, the cost of gene editing therapies is likely be even more expensive.^2^^,^^3^ Indeed, companies that are working on gene editing therapies are pricing the therapy at around $2 million to $3.5 million per patient, arguing that a curative one-time gene editing treatment will save the health care system years of costly imaging studies, procedures, and hospitalizations. While this may be true, it is reasonable to ask whether gene editing therapies will really save the health care system $3 million dollars per patient when compared with the current standard of care. For example, 30 years of therapy with simvastatin costs ∼$11,000, and reduces hospitalizations by 20%.^4^ Nonetheless, it should be recognized that as many as 50% of patients who start statin therapy will stop taking their statin within the first year.^5^ Treatment with Inclisiran, which is a long-acting, short-interfering RNA (siRNA) that selectively targets proprotein convertase subtilisin/kexin type 9 synthesis in the liver and is given twice per year, costs <$9,000 per year, thus overcoming some of the inherent problems with the statin tolerability and noncompliance.^5^ Although economies of scale may lower the projected costs of treatment with gene editing strategies for treating common cardiovascular diseases, they are unlikely to reduce the cost to the cost levels for current U.S. Food and Drug Administration (FDA)–approved therapies for treating atherosclerotic cardiovascular disease.

Beyond the projected costs for gene editing therapies currently under development is another important societal issue: whether this potentially life-saving therapy can also be developed to treat patients with rare diseases. As highlighted in a recent paper in the *New York Times* by Fyodor Urnov, a Professor of Molecular and Cell Biology at the University of California, Berkeley, for diseases with fewer than 100 patients, the prices for gene therapy medications are frequently insufficient for small companies to sustain commercial viability. Urnov cites the example of bluebird bio, a biotechnology company that successfully developed a gene therapy for sickle cell disease and is struggling to remain financially viable, despite having an FDA-approved therapy that is priced at $3 million.^3^ For gene editing technology to fulfill the promise of personalized medicine, it will need to be available to all patients afflicted with rare and common cardiovascular diseases, which means that it also needs to be financially feasible to develop and sustain. Recognizing that it is unlikely that biotech companies will be willing to invest $10 million dollars over a period of 3 to 4 years to develop a cure for a disease that affects a small number of patients, what are some possible solutions to overcome this financial inertia? Urnov suggests that the FDA should develop a more streamlined regulatory process for bringing CRISPR medicine tailored to patients with rare diseases. Here is where the experience gained with CRISPR technologies that are being developed to treat common cardiovascular diseases may be brought to bear and provide insights into streamlining the regulatory process for less common diseases. However, this is unlikely to completely overcome the problem. In countries where health care is centralized, governments may choose to negotiate prices with companies before granting approval for the new gene editing technology. Although financially perspicacious, this approach raises the thorny question of whether taxpayers should be asked to pay for therapies that affect <1% of the population. Another suggestion that has been proposed is for international collaborations to help support expensive new therapies. For example, the Bill and Melinda Gates Foundation is collaborating with Novartis and the National Institutes of Health to identify strategic partners in sub-Saharan Africa to foster basic and clinical gene therapeutic approaches in this region.^2^

For gene editing strategies to truly benefit humanity, this exciting technology will need to be developed for patients with rare and common diseases who live in developed and developing countries. Although the path forward is not clear at this time, it will require the collaborative efforts of drug companies, regulatory agencies, funding agencies, patient advocacy groups, academic consortia, and policy makers to fulfill the promise of this revolutionary new technology. As always, I welcome your thoughts about the myriad of issues related to gene editing for cardiovascular disease, either through social media (*#JACC:BTS*) or by e-mail (jaccbts@acc.org).

## References

[1] Gillmore J.D., Taubel J., Gane E. First-in-human in vivo CRISPR/Cas9 editing of the TTR Gene by NTLA-2001 in patients with transthyretin (ATTR) amyloidosis with cardiomyopathy. AHA 2022 Presentation Slides. American College of Cardiology. https://www.acc.org/education-and-meetings/image-and-slide-gallery/media-detail?id=44bd7ca7119744aab5305f8f67fbb10c.

[2] (2021). Gene therapies should be for all. Nat Med.

[3] Urnov F. We can cure disease by editing a person’s DNA. Why aren’t we? *New York Times*. https://www.nytimes.com/2022/12/09/opinion/crispr-gene-editing-cures.html.

[4] Heart Protection Study Collaborative Group (2009). Statin cost-effectiveness in the united states for people at different vascular risk levels. Circ Cardiovasc Qual Outcomes.

[5] Desai N.R., Campbell C., Electricwala B. (2022). Cost effectiveness of inclisiran in atherosclerotic cardiovascular patients with elevated low-density lipoprotein cholesterol despite statin use: a threshold analysis. Am J Cardiovasc Drugs.

